# A Facilitated Web-Based Self-Management Tool for People With Type 1 Diabetes Using an Insulin Pump: Intervention Development Using the Behavior Change Wheel and Theoretical Domains Framework

**DOI:** 10.2196/13980

**Published:** 2020-05-01

**Authors:** Claire Reidy, Claire Foster, Anne Rogers

**Affiliations:** 1 National Institute for Health Research Collaboration for Leadership in Applied Health Research and Care School of Health Sciences, Faculty of Environmental & Life Sciences University of Southampton Southampton United Kingdom; 2 School of Primary Care, Population Health & Medical Education Faculty of Medicine University of Southampton Southampton United Kingdom; 3 Macmillan Survivorship Research Group School of Health Sciences, Faculty of Environmental & Life Sciences University of Southampton Southampton United Kingdom

**Keywords:** web-based intervention, behavior change wheel, type 1 diabetes, social support, continuous subcutaneous insulin infusion, self-management

## Abstract

**Background:**

Type 1 diabetes (T1D) requires intensive self-management (SM). An insulin pump is designed to better support personal T1D management, but at the same time, it exacerbates the complexity and requirements of SM. Research shows that people with diabetes are likely to benefit from navigating and connecting to local means of social support and resources through web-based interventions that offer flexible, innovative, and accessible SM. However, questions remain as to which behavior change mechanisms within such resources benefit patients most and how to foster engagement with and endorsement of SM interventions.

**Objective:**

The aim of this study was to evaluate the perspectives and experiences of people with T1D using an insulin pump and specialist health care professionals (HCPs) and determine what behavior change characteristics and strategies are required to inform the optimization of an existing web-based social network (SN) intervention to support SM.

**Methods:**

Focus groups with insulin pump users (n=19) and specialist HCPs (n=20) in 6 National Health Service (NHS) trusts across the south of England examined the barriers and enablers to incorporating and self-managing an insulin pump. An analysis was undertaken using the Behavior Change Wheel and Theoretical Domains Framework, followed by a taxonomy of behavior change techniques (BCTs) to identify the contents of and strategies for the implementation of a complex health intervention.

**Results:**

A total of 4 themes represent the SM perspectives and experiences of stakeholders: (1) a desire for access to tailored and appropriate resources and information—the support and information required for successful SM are situational and contextual, and these vary according to time and life circumstances, and therefore, these need to be tailored and appropriate; (2) specific social support preferences—taking away isolation as well as providing shared learnings and practical tips, but limitations included the fear of judgment from others and self-pity from peers; (3) the environmental context, that is, capacity and knowledge of pump clinic HCPs—HCPs acknowledge the patient’s need for holistic support but lack confidence in providing it; and (4) professional responsibility and associated risks and dangers, whereas HCPs are fearful of the consequences of promoting non-NHS SM support, and they question whether SM support fits into their role. BCTs were identified to address these issues.

**Conclusions:**

The use of behavioral theory and a validated implementation framework provided a comprehensive approach for systematically identifying barriers and enablers of self-managing T1D with an insulin pump. A web-based SN intervention appears to offer additional forms of SM support while complementing NHS services. However, for intervention implementation, HCPs’ apprehensions about responsibility when signposting to non-NHS SM support would need to be addressed, and opportunistic features would need to be added, through which pump users could actively engage with other people living with T1D.

## Introduction

### Background

In the United Kingdom, approximately 400,000 people are currently living with type 1 diabetes (T1D), and both the prevalence of T1D and the health care costs of managing T1D are increasing [1,2]. An improvement in blood glucose levels is viewed as a primary goal of self-management (SM) efforts, as it delays the onset and progression of diabetes-related complications (stroke, heart disease, and neuropathy). However, only 30% of the people with T1D are achieving the recommended glycemic targets [3], and attaining these targets is complex. There is recognition of the need for more tailored interventions to enhance the opportunity to improve blood glucose levels [4]. Theoretically founded web-based interventions in particular are seen to offer the opportunity to support flexible, innovative, and accessible SM to address this growing crisis [5].

### The Complexities of Treating and Managing Diabetes

Treatment of T1D comprises demanding SM requirements, including insulin therapy (multiple daily injections [MDI] or insulin pumps); self-monitoring of blood glucose; and comprehensive understanding of nutritional, hormonal, and physical impacts on glycemia [6,7]. MDI is the most common insulin therapy method, but interest in and uptake of insulin pumps have risen over the past 20 years, and predictions suggest that this will continue because of the growing global interest and evidence supporting their use [8]. The Diabetes Attitudes, Wishes, and Needs second study (DAWN2) found that the outcomes are better for people with diabetes when they have greater access to diabetes SM education and positive social support [9]. A recent review suggested that interventions to improve these aspects are necessary and require more flexible and personal SM support for those using these devices [10]. The review highlighted how the process of incorporating an insulin pump often changes treatment expectations and experiences and comprises a distinct and potentially difficult process of learning, exploration, and adaptation. People with T1D initiating a new health technology need to self-manage, but they need appropriate options to do so, and web-based interventions have unlocked potential in this regard.

### New Approaches to Self-Management of Type 1 Diabetes

Technology can play a key role in bringing diabetes care to the individual [11]. Interest in web-based SM interventions has increased over the past decade [11,12], as web-based elements (or electronic health) offer opportunities to take pressure off the National Health Service (NHS) while supporting flexible and accessible SM [5]. In addition, interventions that take into account the individual’s social context in behavior change are relevant in improving health outcomes [13]. It is well recognized that poor psychological well-being can have a significant impact on glycemic control, which consequently increases the risk of diabetes-related complications and leads to increased health care costs and lost productivity [11,14-18]. Increased valued social involvement is linked to greater SM capacity and potentially lower formal health care costs, especially when this involvement is from a diverse set of network members [19,20]. Network members can be health care professionals (HCPs), family, friends, colleagues, community groups, objects (eg, a bicycle), pets, and spiritual groups. Social networks (SNs) and good social support have been shown to promote diabetes SM and assist in physical and mental well-being [21-27]. An SN approach focuses on available and underused collective support from network members as well as on behavior change at a cognitive level [21-27]; therefore, a web-based social support network intervention could provide a currently underutilized avenue for improved psychological well-being and blood glucose levels.

Blakeman et al’s [28] randomized controlled trial of an early version of the Generating Engagement in Networks InvolvEment (GENIE) SN intervention demonstrated improved quality of life, engagement in health care, and health outcomes. GENIE is a web-based SN tool that provides SM support by helping participants map their personal community of support and make the best use of existing contacts and add new ones where needed, as well as signposting (and providing a nudge) to personalized resources in their locality [29]. Despite this demonstration of success, little progress has been made in implementing and spreading psychosocial or social support interventions, in general, into clinical practice to improve SM [6,30-33]. There are challenges in the implementation, sustainability, and accessibility of these interventions in local contexts and to relevant stakeholders (patients and HCPs) [34]. The consideration of the mechanisms of success is often missing [35]. For example, Mulvaney et al’s [36] review of the diabetes mobile intervention design found that there was often little consideration for what SM barriers were addressed or the likely motivation for potential users. They suggested tailoring health intervention content and design (such as GENIE) to stakeholder characteristics to improve patient engagement and outcomes.

The Medical Research Council has identified the importance of utilizing theory and incremental stepped approaches when developing behavior change interventions [37]. In this instance, the Behavior Change Wheel (BCW) and the Theoretical Domains Framework (TDF) were selected because of their focus on the context (the physical setting) in which a behavior occurs, the reflective processes that are involved in behavior change, and the provision of a clear and direct strategy to foster change [38-40]. The evidence base for digital SM interventions in long-term conditions may be able to progress more effectively if we not only focus on measured outcomes but also document and examine the dimensions and processes of interventions most important to stakeholders.

This paper provides a comprehensive needs identification of the specific insulin pump SM needs and perspectives of people with T1D and HCPs working in T1D pump clinics. This will identify recommendations to adapt and optimize the preexisting web-based intervention GENIE, both in terms of the content of the existing intervention and the implementation of the intervention with the aim of improving the SM of people with a long-term condition (such as diabetes) while implementing a new health technology (such as an insulin pump) from the point of technology initiation.

## Methods

### Study Design

This qualitative study consisted of focus groups, which provided the opportunity to explore the range of views and perspectives of the support required and resources used by current pump users, from pump initiation to current point of use. Focus groups were used as a means to facilitate discussion [41], and focus groups are known to stimulate enhanced disclosure and a supportive environment, which incites elaborated accounts and clarification of experiences [42]. Focus groups with HCPs allowed for the exploration of how a web-based SM support tool could fit into NHS practice. The group environment was considered a strength for discussions of implementation and offered an opportunity for individual HCPs to respond to and build on colleague’s comments and brainstorm ideas. Focus groups were undertaken until “saturation” (ie, no significant new insights emerged) [43].

### Ethics

Ethical approval for this study was granted by the University of Southampton (Reference 26208) and the National Research Ethics Service (Reference 17/NS/0089).

### Setting

The study took place between July 2017 and January 2018 in the south of England. The focus groups took place within 6 NHS trusts, which represented varying levels of deprivation and population density across the region.

### Population Sample

Pump user participants were purposefully sampled to ensure a range of pump user ages, length of diagnosis, marital status, sex, and employment status to reflect differing perspectives. Clinics were purposively sampled to represent natural variation across different secondary care settings (urban/semirural, varying deprivation levels, and commissioning procedures). Participants in the focus groups held an advisory capacity for the adaption of an SN SM intervention; therefore, variation was prioritized to improve the likelihood of the resulting intervention being fit for purpose, and it was developed appropriately according to the needs of a variety of pump users and within the context of secondary care.

Eligible patient participants were aged 18 to 65 years, had been diagnosed with T1D for more than 1 year, and had an insulin pump for more than 6 months. Participants who had lived with a pump for less time were excluded to focus on the experiences of overcoming, and reflection of, the initial period of adjustment. Diagnosis of diabetes for less than 1 year was also excluded so as to not obscure the experiences of incorporating a new technology with those of a new diagnosis. Participants were invited to take part through social media, posters in local pump clinics, local diabetes charities, and peer support groups.

All HCPs in insulin pump clinics working directly with patients were eligible to participate in the study and were invited to attend focus groups through direct contact with the clinic.

### Theory

**Figure 1:**
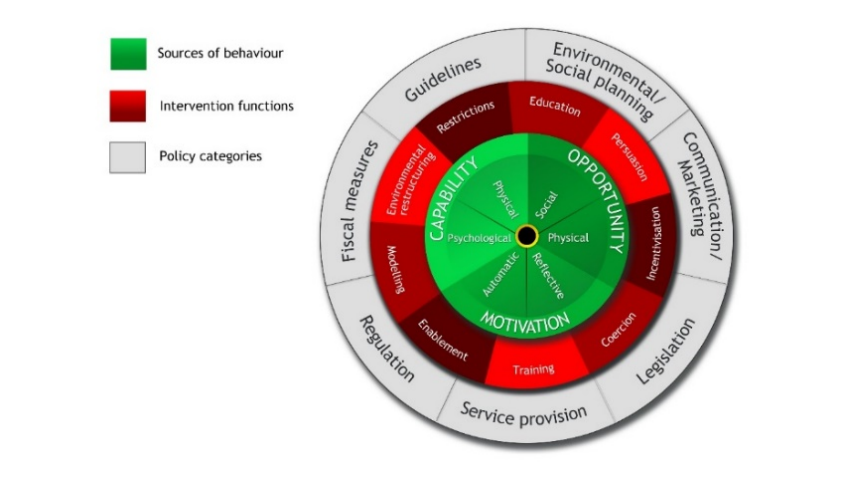
Determining the potential mechanisms of action of an intervention using the Behavior Change Wheel.

**Figure 2:**
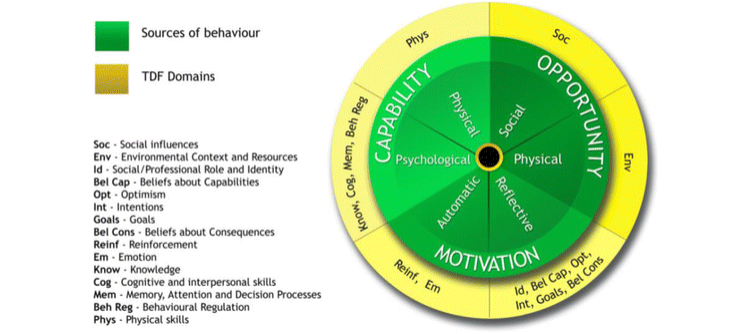
The 14 domains of the Theoretical Domains Framework, structured according to the Capability, Opportunity, Motivation-Behavior model. TDF: Theoretical Domains Framework; Soc: Social influences; Env: Environmental context and resources; Id: Social/professional role and identity; Bel Cap: Beliefs about capabilities; Opt: Optimism; Int: Intentions; Bel Cons: Beliefs about consequences; Reinf: Reinforcement; Em: Emotion; Know: Knowledge; Cog: Cognitive and interpersonal skills; Mem: Memory, attention, and decision processes; Beh Reg: Behavioral regulation; Phy: physical skills.

**Figure 3:**
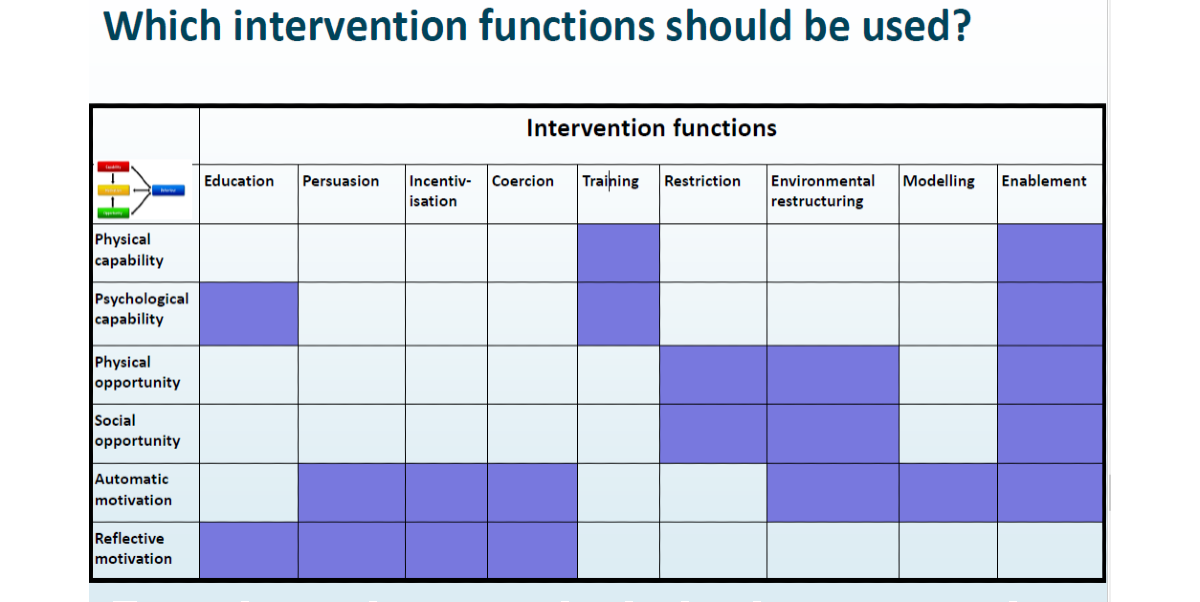
Intervention function mapping matrix.

The BCW [39] is an overarching framework from a synthesis of behavior change interventions providing a clear all-encompassing model of behavior change (see Figure 1). This synthesis integrates theoretical constructs leading to successful behavior change in a variety of health settings. The central cog of the BCW comprises the Capability, Opportunity, Motivation-Behavior (COM-B) components (see Figure 2). This is based on the premise that to initiate behavior change, there is a need to maximize physical or psychological “Capability” to regulate behavior (ie, develop relevant skills), increase or decrease automatic or reflective “Motivation” to engage in desired/undesired behavior, and target the physical or social “Opportunity” to support behavior change. The COM-B offers an understanding of the barriers and enablers of behavior and underscores the potentially modifiable factors for an intervention to target. The BCW links the COM-B model results with intervention functions (see Figure 3). We also utilized the TDF [38,44] (see Figure 2) to provide specific and comprehensive behavioral domains to target in the intervention. The TDF compounds 84 constructs from multiple psychological theories (motivational, action, and organizational theories) and comprises 14 domains of theoretical constructs [44-46]. The TDF provides a useful framework for understanding the barriers and factors influencing specific behaviors [44,47,48]. It provides a detailed analysis of the potentially modifiable factors linked with the BCW (the COM-B components in the central cog of the wheel) to target in an intervention. For example, if lack of knowledge prevents SM, this would be coded as “psychological capability” in COM-B; thereafter, more specifically, “Knowledge” using the TDF and the intervention function mapping of the BCW might suggest an intervention function of “education.” Using the BCW and the TDF in this way has been recommended elsewhere [38,44,47].

A taxonomy of behavior change techniques (BCTs) [49] then enables the specification of techniques describing the active components of the intervention to tailor and optimize an SN intervention. Focus group interview topic guides for both patients and HCPs were developed in consideration of the components of the COM-B model [39] and TDF [44] to ensure participants had the opportunity to explore each element (eg, physical opportunity to self-manage).

### Behavioral Analysis

**Figure 4:**
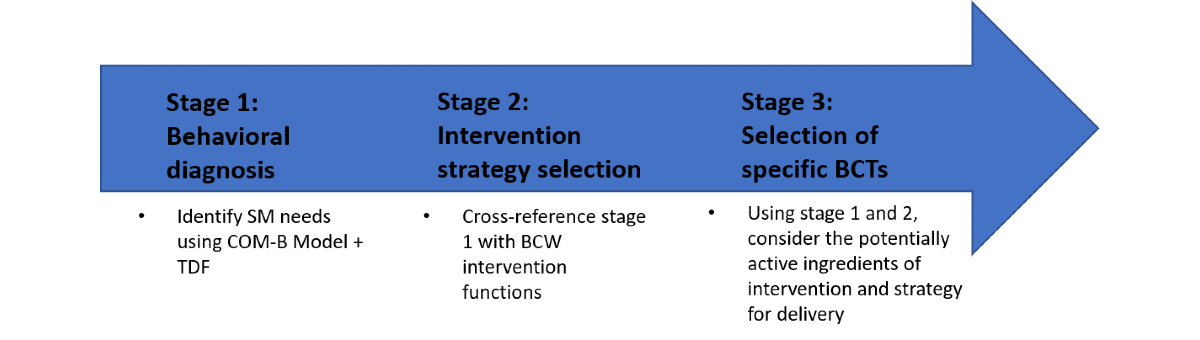
Determining the potential mechanisms of action of an intervention using the Behavior Change Wheel. BCTs: behavior change techniques; SM: self-management; COM-B: Capability, Opportunity, Motivation-Behavior; TDF: Theoretical Domains Framework; BCW: Behavior Change Wheel.

Behavioral analysis using the BCW and TDF and complimentary taxonomies of BCTs comprised 3 stages to systematically determine the necessary mechanisms of action for supporting SM and developing a suitable intervention (see Figure 4).

#### Stage 1: Behavioral Diagnosis

The first stage identifies the behaviors identified in the focus groups in context of using the COM-B model as a framework (stage 1a), broken down into physical or psychological capability, reflective or automatic motivation, and social or physical opportunity and determined as barriers and enablers. The COM-B model was then used alongside the TDF to provide a more comprehensive behavioral analysis by allocating more specific behavioral domains to focus on within the intervention (or delivery of the intervention, eg, a focus on addressing knowledge or skill; stage 1b). All focus group interviews were transcribed verbatim. A deductive approach to analysis was used for the initial analysis, using the theoretical framework provided by the COM-B model and the TDF [50]. The data were further analyzed inductively (by CR) to identify the overarching themes within the COM-B and TDF subcomponents to summarize quotes representing similar underlying ideas (see Figure 3) [51].

#### Stage 2: Intervention Strategy Selection

The second stage cross-references the behavioral diagnosis (stage 1a and 1b) with the BCW “intervention functions” (“education,” “persuasion,” “incentivization,” “coercion,” “training,” “restriction,” “environmental restructuring,” “modeling,” and “enablement”; see Figure 1).

The intervention mapping matrix (Figure 3) was employed to establish which intervention functions would be most pertinent in targeting the SM support required.

#### Stage 3: Selection of Specific Behavior Change Techniques

Stage 3 identified the BCTs that would be required in the facilitated web-based SN intervention—GENIE (components of the intervention such as goal setting, restructuring the social environment, and framing/reframing) [49]—according to the findings of stages 1 and 2. This allowed us to determine the necessary mechanisms of action for SM support intervention (GENIE) and to which the Acceptability, Practicability, Effectiveness/Cost-effectiveness, Affordability, Safety, Equity (APPEASE) criteria were applied. The APPEASE criteria provide guidelines to consider for the content and delivery of the intervention, based on affordability, practicability, effectiveness and cost-effectiveness, acceptability, side-effects/safety, and equity considerations. In addition, a distinction was made regarding both the potentially active ingredients of the intervention (named “Reflective” processes) and the components and delivery of the intervention (context/setting; named “Strategic” processes).

## Results

### Results of Focus Groups

A total of 11 focus groups and 1 interview were carried out; 6 focus groups were carried out with insulin pump users (n=19; see Table 1), and 5 focus groups and 1 interview were carried out with diabetes specialist HCPs (n=20). We held 1 focus group per clinic (except one where we also undertook an interview). Conversations lasted from 40 to 72 min (average=56.33 min) with patients and 27 to 44 min (average=37.6 min) with HCPs.

**Table 1:** Participant demographics.

Characteristics		Values
Age (years), mean (SD); range		38.53 (9.91); 20-53
Sex (female), n (%)		10 (53)
Ethnicity (white British), n (%)		16 (84)
**Income^a^, n (%)**		
	Lower than average	8 (42)
	Average	6 (32)
	Higher than average	5 (26)
Education level (degree level or above), n (%)		12 (63)
Time since diagnosis (years), mean (SD); range		21.95 (12.77); 3-41
Time since pump start (years), mean (SD); range		5.94 (5.98); 0.5-19
Diabetes-related complications^b^, n (%)		9 (47)
Been in hospital >3 times^c^ for hypoglycemia or diabetic ketoacidosis, n (%)		2 (11)
Health care professionals, n		
**Role, n (%)**		
	Diabetes specialist dietician	5 (25)
	Diabetes specialist nurse	7 (35)
	Diabetes consultant	7 (35)
	Diabetes assistant practitioner	1 (5)
Sex (female), n (%)		15 (75)
Age (years), (%); range		70; 45-54
Ethnicity (white British), n (%)		16 (80)
Time in diabetes clinical practice, mean (SD); range		13.69 years (8.22); 2 months-27 years
Time working with pumps, mean (SD); range		8.74 years (5.98); 2 months-24 years
Time working in current diabetes clinic, mean (SD); range		10.11 years (7.62); 2 months-25 years

^a^Average income in the United Kingdom=£26,500.

^b^Eye damage: background retinopathy/eye damage/treated retinopathy/nerve damage (neuropathy)/other complications.

^c^Over the last 3 years.

#### Behavioral Analysis Stage 1 Results: Framework Analysis

The findings of the framework analysis providing a matrix of links among the COM-B model and the TDF are presented in Multimedia Appendices 1 and 2.

#### Stage 1 Results: Thematic Analysis

A total of 4 key themes were identified from the thematic analysis of transcripts; data and quotes are presented to illustrate each theme rather than theoretical subcomponent for conceptual accessibility: (1) desire for access to tailored and appropriate resources and information, (2) specific social support preferences, (3) the environmental context—capacity and knowledge of pump clinic HCPs, and (4) professional responsibility and associated risks and dangers.

#### Desire for Access to Tailored and Appropriate Resources and Information

It was acknowledged that at the initiation of pump therapy, the pump can be complicated and difficult to master. Patients reported a desire for holistic support and flexible, convenient access to information and resources as well as access to the latest scientific research, but only at a time suitable for them (as and when). Web-based support was particularly salient because of ease of access. This kind of support, information, and resource was desired in times of heightened difficulty and situational change (eg, pregnancy, health complications, new employment arrangements, and experience of “burnout”). People’s time was also limited; therefore, resources had to be used wisely, both in terms of attending clinics and accessing assistance. All the pump user focus groups included substantive discussions about access to tailored and advanced fitness-related information. Performing exercise along with others living with T1D or seeking advice from others about exercise were expected to ease some of the anxieties about experiencing (or preparing for) low (or high) blood glucose levels during exercise:

I don’t know if any of you have heard of the website Runsweet or Ex-carbs or anything like that?...All of the rest of the Type 1 diabetes management was fine for me, but exercise was my big issue...Anyway, Ex-carbs is a website that helps you to come up with a good way to begin exercising.

Dan, pump user

In addition, relevant information was needed, which was specific to T1D and/or insulin pumps, rather than general information for any type of diabetes:

It would be nice to have access to a website that gives you information about diets and Type 1 diabetes. I go to [diabetes charity], but it's not up-to-date. It's for Type 2.

Katherine, pump user

Access to other holistic pursuits were cited as important, owing to the participants’ desire for enjoyable activities for the promotion of positive mental health and/or finding that these activities also required some navigation in terms of the impact on their glycemic control:

I’ve never been really sporty...I also do get a little bit annoyed that every time anyone [in diabetes groups] does talk about any kind of social interactions, other than “meet-ups”, it’s always revolved around sports. I would love to see, or even run, some more diabetic-friendly groups that are, for example, theatre based. The pressure of being on stage is likely to cause hypos or have a high so you need a group which understands that, you know?

Stephanie, pump user

#### Specific Social Support Preferences

Social support was fundamental to most insulin pump users. Flexible and open contact with the clinic was valued, although this did depend on personal experiences with HCPs, but support from peers was equally valued. Being among other people with T1D, both on the web and offline, provided a wealth of otherwise unseen yet vital information for day-to-day life, such as practical tips and provision of assistance (faulty equipment and where to place the pump on the body). This need varied according to circumstance: T1D-specific support groups, especially if newly diagnosed, were desired, and diabetes-specific fitness groups were valued for the opportunity to determine how best to exercise without glucose levels rising too high or falling too low or how/where to carry the extensive equipment. Meeting peers was associated with taking away some of the isolation of living with a hidden condition:

[I would like] social things like groups that you can meet people who are in a similar situation to you...because you can’t just walk down the street and ask “are you on a pump?”

Mark, pump user

But actually I had no idea that diabetes-- I remember thinking this condition was incredibly rare, because I never knew anyone else with it.

Jenny, pump user

Access to peer support was cited as important in sharing stories, troubleshooting, sharing illness burden, and speaking to people who understand this “invisible” condition. Some desired web-based support, whereas others desired face-to-face contact, and it was common to desire both. Although face-to-face interactions were important, web-based access allowed people to conveniently “dip in” or “lurk” from a safe distance. In addition, participants expressed wanting to be of assistance themselves, providing support of mutual (reciprocal) benefit. However, apprehensions were raised about accessing people who were in a similar situation. Identities began to be focused on the basis of being pump users:

Personally, I find having a one-on-one conversation with someone and asking questions...as wonderful as the nurses are, and the clinic nurses are fantastic, but having someone who uses a pump every single day- It was really positive being in a group setting and having conversations amongst ourselves...You could say “what do you do while you're asleep?” “Do you ever get over having something strapped to you?” Just basic questions.

Harry, pump user

I guess more links...I had some like issues with it [the pump] sticking on--and no one’s ever told me about what kind of tapes that I can use to keep it on or stuff like that, or even nice covers for your pump, just like nice things that are easier to find through that [social-network intervention] rather than having to go through Amazon.

Lauren, pump user

There were distinct barriers to speaking to others with T1D, such as a lack of confidence, especially when there was a perceived risk of peer judgment or competition:

Because if you are nervous of -- If you don't have the best control or you have been through a bit of a rough patch, or you don't really know-you know-It must be daunting to meet other people so I think you have to be in the right kind of place to want to—

Jenny, pump user

#### The Environmental Context: Capacity and Knowledge of Pump Clinic Health Care Professionals

Many HCPs were positively encouraging of the psychosocial needs of patients and recognized that social and peer support were valuable for patients:

Yes, so, it is useful. It's very positive. The good thing I like about it is the opportunity to meet other people, network and do other things outside of diabetes, and for them to feel as normal as possible, but they are normal. You know what I mean?

Diabetes Specialist Nurse 5, HCP

HCPs were enthusiastic about supporting their patients to self-manage, especially in terms of patient’s need for holistic support and resources, but they lacked confidence in addressing the psychosocial needs of patients themselves:

I think it’s a question of whether we think we’re skilled. I think it’s more a part of taking history but it realms into the psychological support, psychology support territory and whether as nurses and dietitians and clinicians, we think we would have the skills to deliver that. I think it’s something which if it was something very, do tick box; A, B and C, this is something which we don’t do in our routine clinical basis...but a lot of the care is focused towards the more technical and medical and other supportive aspects.

Consultant 4, HCP

However, some HCPs expressed a lack of value for psychosocial support or SM support where it was not seen as part of their clinical remit:

So, realistically...resources that are available are something that you kind of say--, “oh look I know I've got my little ‘talking change’ thing” and my “little thing in there for somebody who” and “that’s a resource that I can make available”, but, I don't say, “Would you like to talk to a psychology person--?” to everybody that comes in…and I suppose that a lot of it is that if it's not broken what’s to fix?

Consultant 6, HCP

Most clinicians were interested in innovative ways for patients to access other support. They were especially enthusiastic about their patient’s needs, with an appreciation of the benefits of engagement with other people with T1D, especially others with a pump, for shared learnings and experiences. Some clinicians considered the potential facilitation of access to social support interventions in structured education sessions, whereas others considered approaches to such support via signposting through their clinic rather than providing access within. However, HCPs were concerned about competing priorities and the consequential lack of time/capacity in the clinic to engage in SM support or offer a facilitated web-based intervention:

...I think the CCG fund the pumps but we don’t have an awful lot of funding for the team that supports the pump service, so whilst we had small numbers we could incorporate it into our service level agreement but as the pump service has grown we’re struggling to offer the support we would like to offer. The feedback we’re getting is our pump patients love our service and want more of it but actually we can’t really give them anymore because we’re not funded to.

Dietician 4, HCP

#### Professional Responsibility and Associated Risks and Dangers

Some HCPs were evidentially concerned about the risk and dangers of signposting or onward referral to a web-based SM support tool, and they held fears that such signposting to a nonclinical environment could have negative consequences in terms of their professional responsibility:

Yes, or, accuracy of...Or the potential dangers of peer-to-peer advice regarding immediate clinical matters. I think that’s my opinion at the moment. Sharing it in a controlled way with the, you know, organizations that are available to have them. In terms of peer-to-peer advice, what if someone gives them the wrong advice? Maliciously, for instance.

Consultant 7, HCP

Some HCPs also felt that this could be “creating problems for problems sake” by offering SM support services within a clinical setting:

My first thought about this, is it bringing up things that we actually don’t need to bring up, I would think that. I know we do want to make sure that everybody is well supported and has access to that support. At the same time, if somebody’s absolutely fine...We don’t want to be making them feel that there is something wrong when there isn’t...What you don’t want to be doing is creating problems. For problems sake.

Dietician 3, HCP

However, pump users referred to unhelpful experiences of HCPs blocking access to information, resources, or medical equipment. Patients demonstrated an understanding of risk, but they also demonstrated the need to make decisions themselves:

Going back to that idea of online groups, I understand that you would want to have a warning to say, “this is not NHS, this is not moderated. This is just a group that is publicly available and we’re not recommending or making any sort of judgment”. I’m fine with the warning but ideally would want to still have a link to it...I understand the caution but one of my pet peeves is when healthcare professionals make a choice for me [agreement in room] and say I’m not going to bother to give you the bigger picture and the different options because I think this one is best for you.

Hugh, pump user

HCPs considered an SN intervention especially useful for patients who were young adults going through transition or any patient experiencing loneliness. However, patients felt that they themselves would benefit from further support, no matter their circumstances, but according to when they needed it and on their own terms.

### Stage 2 Results: Intervention Strategy Selection

#### Relevant Intervention Functions for Pump Users

##### Capability

Psychological capabilities were identified in the behavioral diagnosis, and using the intervention mapping matrix, (Figure 3) the following intervention functions were identified: Enablement (a means to increase capability or reduce barriers for SM through encouragement, practical and emotional support, and access to support and opportunities) and Education (increasing knowledge or understanding, including structured education, access to appropriate information, and instructions for performing pump tasks). Physical capability SM barriers and enablers pinpointed to the intervention functions of Training (imparting physical skills in relation to pump technicalities) and Enablement (as described above).

##### Motivation

Motivational factors of SM that were related to reflective reasoning (conscious intentions, decisions, and plans for SM) led to the intervention functions of Education (as described above). Where there were reflective motivational barriers preventing SM because of support not being seen as relevant or an intervention not being credible, then the intervention functions such as Persuasion through communication to introduce positive feelings to stimulate action or assurance of credibility through research were selected. Where an SN intervention enables SM with self-driven priorities, it increases the likelihood that users will be willing to commit time and that the time they commit will be well spent and valued. Appropriate intervention functions for automatic motivation for SM (emotional responses, desires, and habits) included Persuasion, Environmental restructuring (changing the physical or social context), Modeling (providing an example for people to emulate/aspire to), and Enablement.

##### Opportunity

Social and physical opportunity to access both emotional and practical support, especially in relation to the specificities and mechanics of a new health technology, was identified in the behavioral diagnosis, and this could be addressed by an SM support web-based intervention. These needs were described in terms of unconventional and flexible ways to self-manage, such as 24/7 access and web-based sources of education, peer support, and information. However, access to any support or resources had to be on the participants’ terms, in line with personal needs and life demands, especially in response to concerns over uninvited sharing of SM strategies from others. This was linked with the intervention functions of Enablement and Environmental restructuring (providing access to support, information, and opportunities). Enablement intervention functions were identified to address physical opportunity barriers, such as lack of time to attend or access the clinic or other resources in relation to sourcing support that is physically closer to the individual.

#### Relevant Intervention Functions for Health Care Professionals

##### Capability

HCPs said that they believe in prioritizing the wider well-being of their patients and want to support SM, but although they were clear about the medical outcomes they must focus on in their professional role, the remit of SM support they should provide was unclear. HCPs voiced concerns over their lack of confidence, ability, or desire to offer SM support. This is where strategic intervention functions of Training, Enablement, and Education benefit, to instruct HCPs on how to facilitate signposting to an intervention, enable behavioral practice, provide verbal persuasion about capabilities, and educate about the importance of SM support.

##### Motivation

When it came to Reflective motivational factors, it is evident that buy-in is needed. Coercion (changing conscious evaluations of the SN approach for SM), Education (increasing knowledge or understanding of the importance of social support for their patients), Persuasion (using communication to stimulate action), and Incentivization (creating an expectation of reward—that patients will benefit from the access to SM support) were deemed as appropriate intervention functions, whereas Enablement, Environmental restructuring, and Modeling (comparisons with other clinics) were identified for automatic motivational factors.

##### Opportunity

Both physical and social opportunity pinpointed to Enablement and Environmental restructuring (provision of physical opportunities and socially acceptable environments to provide SM support) as necessary intervention functions (see Multimedia Appendix 2).

### Stage 3 Results: Selection of Specific Behavior Change Techniques

The BCTs identified as likely to benefit the intervention are shown in Multimedia Appendices 1 and 2, with the distinction made between the potentially active ingredients of an intervention (“Reflective” BCTs), which would need to be contained within the SN tool, and the delivery of the intervention (“Strategic” processes), which would need to be integrated into the intervention implementation plan, demonstrated in Table 2. Table 2 also describes where or with whom the intervention reflective and strategic processes are to be implemented. These intervention “ingredients” are categorized as in terms of being; the role of the facilitator, an intervention function, within the study protocol, as an invitation to take part, within a site initiation visit, in training, or in future research. This addresses the varying needs and expectations of insulin pump users and HCPs, and this suggests how a web-based intervention designed to enable SM support can attend to these.

**Table 2:** Identified behavior change techniques of intervention (reflective or strategy processes).

Identified needs (behavior change techniques)		SN^a^ intervention ingredients		Where change is to be implemented/delivered	
**Reflective processes**					
	Goal setting (behavior)		Agreement to attend a preferred activity identified in the intervention		Role of facilitator
	Problem solving		An SN tool maps the participants social support network and examines whether the participant would like this to change at all. The intervention also inquires about their personal needs and preferences and then offers opportunities in their local community to address these needs. A discussion is then undertaken about how to access these, as well as barriers and facilitators		Intervention function and Role of facilitator
	Feedback on behavior		The facilitator follows-up with the participants and discusses and informs them of how their circles have changed and what activities have been taken up		Role of facilitator
	Social support (unspecified)		GENIE^b^ facilitates discussion around who offers them social support in relation to their condition and allows facilitation/gives information about further personalized social support, that is, peer support groups, and asks who may help them participate in chosen activities		Intervention function and Role of facilitator
	Social support (practical)		Discuss the practical support required, received, and desired from the participant and facilitate discussion over whether any changes are required and how to undertake these changes or discuss how existing members of the participant’s SN can help them physically access groups		Intervention function and Role of facilitator
	Social support (emotional)		Discuss the emotional support required, received, and desired from the participant and facilitate discussion over whether any changes are required and how to undertake these changes or discuss how existing members of the participant’s SN can help them feel emotionally able to access groups		Intervention function and Role of facilitator
	Instruction on how to perform a behavior		If a person wants to attend a course or education session, then GENIE can facilitate access to this, or if a person wants to learn from peers, then GENIE can point them in the direction of a peer support group		Intervention function
	Prompts/cues		GENIE comprises concentric circles, which prompt the participant to prioritize certain SN members over others. GENIE then asks 13 preference questions to prompt the user regarding the user’s preferred activities to support SM^c^. Participants are then followed up by a facilitator after 2 weeks		Intervention function
	Comparative imagining of future outcomes		Prompt the participant to imagine and compare likely or possible outcomes following attending versus not attending particular groups or activities in which they took part		Role of facilitator
	Reduce negative emotions		The facilitator advises to use members of the current social support network to reduce anxiety about attending groups		Role of facilitator
	Conserving mental resources		The facilitator advises to utilize the social support network or access peer support groups to share the burden of diabetes or to find someone to troubleshoot with		Role of facilitator
	Restructuring the physical environment		Enabling access to groups and information that can help them engage in SM		Intervention function
	Restructuring the social environment		Enabling access to and restructuring groups, information, and support that can help them engage in SM		Intervention function
	Framing/reframing		The facilitator reassures participant that it is okay to ask for help or support from others regarding SM and that others can offer practical tips		Role of facilitator
	Focus on past success		The facilitator enquires about activities they used to do and whether the network members can assist their attendance at activities in which they are interested		Role of facilitator
**Strategy processes**					
	Action planning		Steps would need to be taken to support each clinic to implement the intervention and identify pathways		Protocol and Site initiation visit
	Review behavior goals		The clinic would need to be reviewed to identify whether further support is required to implement the intervention		Protocol and Continuous communication from the research team
	Behavioral contract		The clinic would need to sign a contract to identify what they expect from the intervention and what support they require		Protocol and Agreements
	Commitment		The clinic would need to make SM support a priority and normalized within the clinic setting and be committed to offering SM support		Site initiation visit and Continuous communication from the research team
	Instructions on how to perform a behavior		Facilitators of GENIE receive training on how to deliver GENIE. The tool currently comes with a training program		Protocol and Training
	Behavioral experiments		Pilot study intervention with clinics to demonstrate intervention benefits in this patient group/context		Future research
	Demonstration of the behavior		Facilitators of GENIE receive training on how to deliver GENIE. The tool currently comes with a training program		Protocol and Training
	Information about others’ approval		Share experiences from other clinics/areas using the tool		Future research and Site initiation visit
	Behavioral practice/rehearsal		Facilitators of GENIE receive training on how to deliver GENIE. The tool currently comes with a training program		Protocol and Training
	Credible source		Buy-in from each area it is applied to is important for implementation. Participants (and HCPs^d^) are assured that GENIE has risen out of former research and that everything put on GENIE is checked		Invitation to take part; Protocol; Site initiation visit; and Training
	Comparative imagining of future outcomes		Prompt the clinic to imagine and compare likely or possible outcomes following implementation of GENIE		Invitation to take part; Protocol; Site initiation visit; and Training
	Restructuring the physical environment		Enabling access to SM support and information that can help patients engage in SM		Invitation to take part; Protocol; Site initiation visit; and Training
	Restructuring the social environment		Enabling physical access to groups and information and support that can help patients engage in SM		Invitation to take part; Protocol; Site initiation visit; and Training
	Framing/reframing		Draw attention to research suggesting that SM support can provide clinical benefits and reduced health utilization. SM support could therefore increase clinic time available rather than decrease clinic time.		Protocol; Site initiation visit; and Training
	Incompatible beliefs		Draw attention to how restricting the provision of SM support is in contrast with national guidance (National Health Service England) which promotes SM support.		Invitation to take part; Protocol; Site initiation visit; and Training

^a^SN: social network.

^b^GENIE: Generating Engagement in Networks InvolvEment.

^c^SM: self-management.

^d^HCP: health care professional.

## Discussion

### Principal Findings

This study provides a model for supporting people who are incorporating a health technology that is new to them (such as an insulin pump), through consideration of key stakeholders’ needs in developing an intervention that aims to provide SM support. This study puts the most important needs at the forefront (stakeholders’ needs), providing evidence of the active components required in a translational web-based intervention. In this instance, the physicality of the pump (the new device) impacts the users’ experience of SM, and the technicalities while using an advanced technology exacerbate SM needs. The specificity of the insulin pump changes people’s priorities, as it impacts their day-to-day experiences and identity. Pump therapy means that users have a renewed need for HCPs, akin to the diagnosis of diabetes, but this need subsides. The pump requires access to a particular network of people for specific troubleshooting needs.

With this in mind, we identified that a long-term condition such as diabetes requires an array of SM approaches and the ability to master these. Utilizing a new health technology or device requires specific skills, understanding, confidence, motivation, and opportunity. The behavioral analysis used here signposted the necessary components of an intervention to support SM. For example, there lies a potential conflict for the person living with T1D, where “good” management takes considerable effort, and this can create a friction between freedom and clinical targets of blood glucose control or the opportunity for tighter control without sacrificing freedom. The question arises as to whether this extra attention will actually improve the quality and length of life. The current SM support options offered to people with a long-term condition such as T1D can incite questions over whether life will be less or more enjoyable if they take part in them, for example, using 5 days of annual leave to attend an NHS structured–education class, not knowing whether this education class will actually be useful. There is a trade-off to be made. If we want to intervene, then we must consider these factors. We found that ultimate behavior change in the SM of diabetes and use of a health technology requires support and resources, the availability of which is personal/specific to the individual and varies according to time and life circumstances. Specific social support can take away some of the work of SM, as well as the isolation, providing shared learnings and practical tips, but limitations include fear of judgment from others and exposure to off-putting self-pity from peers.

We found that an intervention would be more successfully implemented if there were opportunities to access SM support and motivation from pump users by access to relevant disease/technology-specific resources and interests. For example, social opportunity needs to be addressed when HCPs do not entrust pump users with the ability to make their own choices or access nonclinical resources or when HCPs lack psychological capability and/or physical opportunity, with HCPs (even if willing) often not having the confidence or capacity in their clinic to amend or enhance their routine or psychosocial care and questioning whether and where SM fits into their role. The recommendations provided here for delivering training to HCPs to facilitate signposting to holistic SM support, enabling behavioral practice, providing verbal persuasion about capabilities, and educating about the importance of SM echo those given in the DAWN2 study [52,53]. Guidelines within the intervention could give assurances to HCPs about what they are signposting to. However, some HCPs’ “if-it-isn’t-broke-don’t-fix-it” attitude highlighted that although NHS England is pushing for more SM support, it is not reaching or convincing to all clinicians on the ground. Fisher et al [54] suggest a clear 3-step framework for diabetes HCPs to support behavior change. The first step requires clinicians to shift their mind-set, moving from a hierarchal model to a more collaborative model, reorienting from information giving to nuances of patient-driven needs.

HCPs can be seen as gatekeepers or blockers of the provision of SM support necessary to manage a complex condition such as diabetes. There was little doubt among HCPs, even those with general concerns, that particular groups of patients would greatly benefit from being signposted to further support that the clinic did not provide. However, the discrepancies show contrasting beliefs between patients and HCPs, where patients themselves considered this access beneficial in a variety of ways, especially in terms of managing fitness activities, general practical advice, or emotional support. Credibility and likelihood of the effectiveness of an intervention are, unsurprisingly, important for both users and those who guard access (clinicians), particularly for clinicians who offer patients the opportunity to participate. Priorities vary depending on perspective, and understanding both perspectives at this stage can inform intervention design and how to determine and ensure credibility.

The SN intervention proposed here (GENIE) is structured around facilitating networks and collective, tailored forms of support through the building of dedicated resources in a database. Whether targeting particular groups or long-term conditions as a whole, a web-based SN tool can accommodate multiple SM needs [55]. However, limitations are evident where access to resources is only as good as the resources that are already in place locally. An SN intervention such as this would also benefit people by addressing the identified need to register collective interests and initiate peer support. For example, having the facility for people to “register their interests” in attending or creating groups in their local area or the ability to connect with others in their local area via the intervention platform.

The behavioral assessment of people with diabetes and HCPs draws parallels with past research. For example, Mulvaney et al’s [36] review concluded that SM interventions in diabetes should integrate technology compounded with human contact for clinical support, as well as motivation and support to change behavior for SM (eg, goal setting and problem solving). In addition, the American Diabetes Association [56] considers behavioral elements such as problem solving, decision making, and providing access to electronic health tools as vital to support SM. A focused SN intervention with integral guided facilitation in place is likely to be sensitive to these needs, combined with participant follow-up from the facilitator. A facilitator also has the potential to provide a favorable supportive element to personalized goals in light of findings that the provision of human support was advantageous in other electronic health interventions [57].

People who are empowered and skilled to self-manage their diabetes have improved health outcomes [6,58,59]; therefore, appropriate and tailored access, as opposed to a one-size-fits-all model, is likely to support improved SM. HCPs need to accept patient priorities and means of information and advocacy [60,61] and understanding the importance of experiential evidence. Some noted factors of success in web-based interventions and acceptability have been the focus on psychosocial experiences, feelings of confidence and reduced fear, the availability outside of clinic hours, up-to-date evidence-based guidance, and access to both peer-generated and professional advice [55,58,59,61-65]. However, understanding the barriers preventing HCPs from supporting SM is fundamental too [61,65]. This comprehensive behavioral analysis provides a complete feedback loop for a web-based intervention, which is better equipped to facilitate ongoing SM, considering the needs and strategies for both sets of stakeholders, and determine how, when, and why SM support interventions can be best utilized.

### Strengths and Limitations

The use of focus groups in this study allows an in-depth discussion and understanding of the collective experiences of SM and of patients’ and HCPs’ views, which would be impossible to explore using quantitative methods, and the use of the BCW and TDF-driven interview scripts provides a well-tested, evidence-based guideline and framework. For example, it has been noted that the automatic addition to the reflective process of motivation to enact behavior on the part of HCPs is often overlooked and is important to enhance the behavioral approaches to implementation [66]. The use of theory-driven intervention development signifies areas of key importance to intervention implementation, both behavioral and reflective needs and contextual factors for implementation, and it is a key process to follow. It sensitizes the research to future intervention needs and considerations across different localities. However, although the proposed BCTs were carefully considered in response to stakeholder needs, these have not been developed in consultation with stakeholders, which future work needs to address to verify BCT feasibility. The recruitment of pump users from various clinics and the involvement of clinics in different settings were important elements of the expected variability among local health systems. Although the participants recruited represented a variety of ages and sex, education attainment, and parenthood, the clinicians represented a good and balanced spectrum of the kinds of professionals working in insulin pump clinics. However, the limitations to the study were that the recruited patients were more likely to be those that were particularly open to discussing personal elements of their diabetes management and willing to sit, in their own time, among a group of peers with the same condition, and therefore would not necessarily represent a number of people living with T1D.

### Future Research and Conclusions

Technology is a means to deal with diabetes, and it opens new ways to manage the condition, but it takes time to master; therefore, appropriate support, skills, and information are crucial. People with T1D have a uniqueness of knowledge about their own body, which challenges professional dominance and creates an invisible barrier wherein despite HCPs possessing sound medical knowledge, they are unsure of what and when to share with their patients. HCPs can be gatekeepers for improving SM or for facilitating access to SM support. They are limited by time constraints and fear of professional responsibility. However, a web-based tool that is person based, appropriate, accessible, and adaptive to local needs, along with a strategic (and theoretically informed) approach, can provide a powerful tool for SM support, which can vastly enhance the support already being provided by HCPs [55,63]. This paper has strived to demonstrate the development of such an intervention. The study is particularly timely in that it coincides with The NHS Long Term Plan from NHS England, January 2019, which promises to expand the provision of digital SM support tools [67]. In addition, there has been a recent drive for the integration of psychosocial support into routine diabetes care [18,21], and this study provides an initial engagement with the factors that would impact how psychosocial support is taken up with HCPs and the priorities for patients. The next phase of development is to integrate these findings into strategic intervention implementation criteria for supporting people to engage in SM with a new device and technology such as an insulin pump.

## Appendix

Multimedia Appendix 1 Matrix of links among the Capability, Opportunity, Motivation-Behavior model, Theoretical Domains Framework domains, intervention functions, and behavior change techniques for pump users.

Multimedia Appendix 2 Matrix of links among the Capability, Opportunity, Motivation-Behavior model, Theoretical Domains Framework domains, intervention functions, and behavior change techniques for health care professionals.

## Abbreviations

APPEASE: Acceptability, Practicability, Effectiveness/Cost-effectiveness, Affordability, Safety, Equity
BCT: behavior change technique
BCW: Behavior Change Wheel
COM-B: Capability, Opportunity, Motivation-Behavior
DAWN2: Diabetes Attitudes, Wishes, and Needs second study
GENIE: Generating Engagement in Networks InvolvEment
HCP: health care professional
MDI: multiple daily injections
NHS: National Health Service
NIHR: National Institute for Health Research
SM: self-management
SN: social network
T1D: type 1 diabetes
TDF: Theoretical Domains Framework
