# Correction: A Facilitated Web-Based Self-Management Tool for People With Type 1 Diabetes Using an Insulin Pump: Intervention Development Using the Behavior Change Wheel and Theoretical Domains Framework

**DOI:** 10.2196/21381

**Published:** 2020-07-30

**Authors:** Claire Reidy, Claire Foster, Anne Rogers

**Affiliations:** 1 National Institute for Health Research Collaboration for Leadership in Applied Health Research and Care School of Health Sciences, Faculty of Environmental & Life Sciences University of Southampton Southampton United Kingdom; 2 School of Primary Care, Population Health & Medical Education Faculty of Medicine University of Southampton Southampton United Kingdom; 3 Macmillan Survivorship Research Group School of Health Sciences, Faculty of Environmental & Life Sciences University of Southampton Southampton United Kingdom

In “A Facilitated Web-Based Self-Management Tool for People With Type 1 Diabetes Using an Insulin Pump: Intervention Development Using the Behavior Change Wheel and Theoretical Domains Framework” (J Med Internet Res 2020;22(5):e13980) the authors noted several errors.

In the Results section of the Abstract, the text was revised from:

“(4) professional responsibility and associated risks and dangers, whereas HCPs are fearful of the consequences of promoting non-NHSSM support, and they question whether SM support fits into their role.”

to

“(4) professional responsibility and associated risks and dangers, whereas HCPs are fearful of the consequences of promoting non-NHS SM support, and they question whether SM support fits into their role.”

In the Discussion section, one sentence has been updated to correct reference citations. The text was revised from:

“In addition, there has been a recent drive for the integration of psychosocial support into routine diabetes care [19,22], and this study provides an initial engagement with the factors that would impact how psychosocial support is taken up with HCPs and the priorities for patients.”

to

“In addition, there has been a recent drive for the integration of psychosocial support into routine diabetes care [18,21], and this study provides an initial engagement with the factors that would impact how psychosocial support is taken up with HCPs and the priorities for patients.”

Multimedia Appendices 1 and 2 contained tracked changes comments. These files have now been replaced and no longer contain the comments.

The corrections will appear in the online version of the paper on the JMIR Publications website on July 30, 2020, together with the publication of this correction notice. Because this was made after submission to PubMed, PubMed Central, and other full-text repositories, the corrected article has also been resubmitted to those repositories

