# Supplementary material for: A Facilitated Web-Based Self-Management Tool for People With Type 1 Diabetes Using an Insulin Pump: Intervention Development Using the Behavior Change Wheel and Theoretical Domains Framework
Source: J Med Internet Res. 2020 May 1;22(5):e13980. doi: 10.2196/13980 (PMC7229530; doi:10.2196/13980)
Supplement: Multimedia Appendix 1 [file jmir_v22i5e13980_app1.docx]

**Multimedia Appendix 1** Matrix of links among the Capability, Opportunity, Motivation-Behavior model, Theoretical Domains Framework domains, intervention functions, and behavior change techniques for pump users.

| Behavioral diagnosis using COM-B^a^—barriers and enablers (*stage 1a*) | | | Theoretical Domains Framework v2 domains linking to COM-B components (*stage 1b*) | Potential intervention functions (s*tage 2*) | Suggested BCTs^b^ (using the 93 BCT taxonomy v2; (*stage 3*) |
| --- | --- | --- | --- | --- | --- |
| **Psychological capability** | | | | | |
|  | **Barrier** | | | | |
|  |  | The pump can be complicated and difficult to master | *Skills: (Skills development)* | Education and Enablement | 1.2 Problem solving; 3.2 Social support (practical); 4.1 Instruction on how to perform a behavior; 12.1 Restructuring the physical environment; and 12.2 Restructuring the social environment |
|  | ***Enabler*** | | | | |
|  |  | Need to know where to access services or information | *Knowledge: (Knowledge of task environment)* | Education and Enablement | 1.2 Problem solving; 3.2 Social support (practical); 4.1 Instruction on how to perform a behavior; 12.1 Restructuring the physical environment; and 12.2 Restructuring the social environment |
|  |  | Want to improve comprehension of impact of exercise on SM^c^ | *Knowledge: (Knowledge about condition)* | Education and Enablement | 1.2 Problem solving; 3.2 Social support (practical); 4.1 Instruction on how to perform a behavior; 12.1 Restructuring the physical environment; and 12.2 Restructuring the social environment |
|  |  | Want help to make choices about SM | *Memory attention and decision processes: (Decision making)* | Education and Enablement | 1.2 Problem solving; 3.2 Social support (practical); 4.1 Instruction on how to perform a behavior; 12.1 Restructuring the physical environment; and 12.2 Restructuring the social environment |
| ***Physical capability*** | | | | | |
|  | ***Enabler*** | | | | |
|  |  | Access to practical tips—how to use the pump’s advanced features and where to place pump on body | *Skills: (Practice/skills development)* | Training and Enablement | 3.2 Social support (practical); 4.1 Instruction on how to perform a behavior; 12.1 Restructuring the physical environment; and 12.2 Restructuring the social environment |
| ***Reflective motivation*** | | | | | |
|  | ***Barrier*** | | | | |
|  |  | HCPs who are bombarded with *risks and dangers* | *Environmental context and resources: (Organizational culture/climate)* | Education and Persuasion | 1.1 Goal setting (behavior); 1.2 Problem solving; 2.2 Feedback on behavior; 6.3 Information about others’ approval; 9.1 Credible source; 9.3 Comparative imagining of future outcomes; 11.2 Reduce negative emotions; 11.3 Conserving mental resources; 15.3 Focus on past success; and 13.2 Framing/reframing |
|  |  | Difficult managing a complex condition but believing that the right information delivered in the right way can make management easier | *Beliefs about capabilities: (Beliefs)* | Education and Persuasion | 1.1 Goal setting (behavior); 1.2 Problem solving; 2.2 Feedback on behavior; 6.3 Information about others’ approval; 9.1 Credible source; 9.3 Comparative imagining of future outcomes; 11.2 Reduce negative emotions; 11.3 Conserving mental resources; 15.3 Focus on past success; and 13.2 Framing/reframing |
|  |  | Understanding that not being able to engage in SM will lead to health complications, but thinking about it all the time will not help either | *Beliefs about consequences: (Characteristics of outcome)* | Education and Persuasion | 1.1 Goal setting (behavior); 1.2 Problem solving; 2.2 Feedback on behavior; 6.3 Information about others’ approval; 9.1 Credible source; 9.3 Comparative imagining of future outcomes; 11.2 Reduce negative emotions; 11.3 Conserving mental resources; 15.3 Focus on past success; and 13.2 Framing/reframing |
|  | ***Enabler*** | | | | |
|  |  | SM support that is relevant/specific | *Social role and identity: (Identity)* | Education and Persuasion | 1.1 Goal setting (behavior); 1.2 Problem solving; 2.2 Feedback on behavior; 6.3 Information about others’ approval; 9.1 Credible source; 9.3 Comparative imagining of future outcomes; 11.2 Reduce negative emotions; 11.3 Conserving mental resources; 15.3 Focus on past success; and 13.2 Framing/reframing |
|  |  | Determined to make pump “work” | *Intentions: (Stability of intentions)* | Education and Persuasion | 1.1 Goal setting (behavior); 1.2 Problem solving; 2.2 Feedback on behavior; 6.3 Information about others’ approval; 9.1 Credible source; 9.3 Comparative imagining of future outcomes; 11.2 Reduce negative emotions; 11.3 Conserving mental resources; 15.3 Focus on past success; and 13.2 Framing/reframing |
|  |  | SM tool must be credible | *Environmental context and resources: (Resources/material resources)* | Education and Persuasion | 1.1 Goal setting (behavior); 1.2 Problem solving; 2.2 Feedback on behavior; 6.3 Information about others’ approval; 9.1 Credible source; 9.3 Comparative imagining of future outcomes; 11.2 Reduce negative emotions; 11.3 Conserving mental resources; 15.3 Focus on past success; and 13.2 Framing/reframing |
|  |  | Want to self-manage well and in a way that suits personal circumstances | *Goals: (Goals (autonomous/controlled)* | Education and Persuasion | 1.1 Goal setting (behavior); 1.2 Problem solving; 2.2 Feedback on behavior; 6.3 Information about others’ approval; 9.1 Credible source; 9.3 Comparative imagining of future outcomes; 11.2 Reduce negative emotions; 11.3 Conserving mental resources; 15.3 Focus on past success; and 13.2 Framing/reframing |
| ***Automatic motivation*** | | | | | |
|  | ***Barrier*** | | | | |
|  |  | Never “having a break” from diabetes | *Memory attention and decision processes: (Cognitive overload/tiredness)* | Persuasion; Environmental restructuring; Modeling; and Enablement | 1.1 Goal setting (behavior); 1.2 Problem solving; 3.1 Social support (unspecified); 3.2 Social support (practical); 3.3 Social support (emotional); 9.3 Comparative imagining of future outcomes; 11.2 Reduce negative emotions; 11.3 Conserving mental resources; 12.1 Restructuring the physical environment; 12.2 Restructuring the social environment; and 13.2 Framing/reframing |
|  |  | Feeling burnt out and not able to engage in SM | *Emotion: (Burnout)* | Persuasion; Environmental restructuring; Modeling; and Enablement | 1.1 Goal setting (behavior); 1.2 Problem solving; 3.1 Social support (unspecified); 3.2 Social support (practical); 3.3 Social support (emotional); 9.3 Comparative imagining of future outcomes; 11.2 Reduce negative emotions; 11.3 Conserving mental resources; 12.1 Restructuring the physical environment; 12.2 Restructuring the social environment; and 13.2 Framing/reframing |
|  |  | Overwhelmed by diabetes and consequently not wanting or able to engage in SM | *Emotion: (Negative affect)* | Persuasion; Environmental restructuring; Modeling; and Enablement | 1.1 Goal setting (behavior); 1.2 Problem solving; 3.1 Social support (unspecified); 3.2 Social support (practical); 3.3 Social support (emotional); 9.3 Comparative imagining of future outcomes; 11.2 Reduce negative emotions; 11.3 Conserving mental resources; 12.1 Restructuring the physical environment; 12.2 Restructuring the social environment; and 13.2 Framing/reframing |
|  |  | Not knowing anyone else with type 1 diabetes | *Environmental context and resources: (Barriers)* | Persuasion; Environmental restructuring; Modeling; and Enablement | 1.1 Goal setting (behavior); 1.2 Problem solving; 3.1 Social support (unspecified); 3.2 Social support (practical); 3.3 Social support (emotional); 9.3 Comparative imagining of future outcomes; 11.2 Reduce negative emotions; 11.3 Conserving mental resources; 12.1 Restructuring the physical environment; 12.2 Restructuring the social environment; and 13.2 Framing/reframing |
|  |  | Only having access to people who have very negative experiences of diabetes/not wanting to speak to others | *Environmental context and resources: (Barriers)* | Persuasion; Environmental restructuring; Modeling; and Enablement | 1.1 Goal setting (behavior); 1.2 Problem solving; 3.1 Social support (unspecified); 3.2 Social support (practical); 3.3 Social support (emotional); 9.3 Comparative imagining of future outcomes; 11.2 Reduce negative emotions; 11.3 Conserving mental resources; 12.1 Restructuring the physical environment; 12.2 Restructuring the social environment; and 13.2 Framing/reframing |
|  |  | Feeling alone/isolated | *Emotion: (Negative affect)* | Persuasion; Environmental restructuring; Modeling; and Enablement | 1.1 Goal setting (behavior); 1.2 Problem solving; 3.1 Social support (unspecified); 3.2 Social support (practical); 3.3 Social support (emotional); 9.3 Comparative imagining of future outcomes; 11.2 Reduce negative emotions; 11.3 Conserving mental resources; 12.1 Restructuring the physical environment; 12.2 Restructuring the social environment; and 13.2 Framing/reframing |
| ***Physical opportunity*** | | | | | |
|  |  | ***Barrier*** | | | |
|  |  | Lack of time to attend or access clinic or other resources of SM | *Environmental context and resources: (Barriers)* | Environmental restructuring and Enablement | 1.1 Goal setting (behavior); 1.2 Problem solving; 3.1 Social support (unspecified); 3.2 Social support (practical); 3.3 Social support (emotional); 7.1 Prompts/cues; and 12.1 Restructuring the physical environment |
|  |  | Not having access to practical tips and information | *Environmental context and resources: (Resources/material resources)* | Environmental restructuring and Enablement | 1.1 Goal setting (behavior); 1.2 Problem solving; 3.1 Social support (unspecified); 3.2 Social support (practical); 3.3 Social support (emotional); 7.1 Prompts/cues; and 12.1 Restructuring the physical environment |
|  |  | Desire to access social support but no physical access/opportunity to access | *Environmental context and resources: (Barriers)* | Environmental restructuring and Enablement | 1.1 Goal setting (behavior); 1.2 Problem solving; 3.1 Social support (unspecified); 3.2 Social support (practical); 3.3 Social support (emotional); 7.1 Prompts/cues; and 12.1 Restructuring the physical environment |
|  |  | SM support not local | *Environmental context and resources: (Resources/material resources)* | Environmental restructuring and Enablement | 1.1 Goal setting (behavior); 1.2 Problem solving; 3.1 Social support (unspecified); 3.2 Social support (practical); 3.3 Social support (emotional); 7.1 Prompts/cues; and 12.1 Restructuring the physical environment |
|  |  | HCPs^d^ withholding choice | *Environmental context and resources: (Organizational culture)* | Environmental restructuring and Enablement | 1.1 Goal setting (behavior); 1.2 Problem solving; 3.1 Social support (unspecified); 3.2 Social support (practical); 3.3 Social support (emotional); 7.1 Prompts/cues; and 12.1 Restructuring the physical environment |
|  |  | Organizational restrictions on peer support services | *Social/professional role and Identity: (Organizational commitment)* | Environmental restructuring and Enablement | 1.1 Goal setting (behavior); 1.2 Problem solving; 3.1 Social support (unspecified); 3.2 Social support (practical); 3.3 Social support (emotional); 7.1 Prompts/cues; and 12.1 Restructuring the physical environment |
|  |  | Organizational restrictions on peer support services | *Social/professional role and Identity: (Organizational commitment)* | Environmental restructuring and Enablement | 1.1 Goal setting (behavior); 1.2 Problem solving; 3.1 Social support (unspecified); 3.2 Social support (practical); 3.3 Social support (emotional); 7.1 Prompts/cues; and 12.1 Restructuring the physical environment |
|  |  | Physical characteristics of the pump | *Environmental context and Resources: (Barriers)* | Environmental restructuring and Enablement | 1.1 Goal setting (behavior); 1.2 Problem solving; 3.1 Social support (unspecified); 3.2 Social support (practical); 3.3 Social support (emotional); 7.1 Prompts/cues; and 12.1 Restructuring the physical environment |
|  |  | HCPs not being accessible | *Social/professional role and Identity: (HCP-patient relationship/communication);*  *Environmental context and resources: (Barriers)* | Environmental restructuring and Enablement | 1.1 Goal setting (behavior); 1.2 Problem solving; 3.1 Social support (unspecified); 3.2 Social support (practical); 3.3 Social support (emotional); 7.1 Prompts/cues; and 12.1 Restructuring the physical environment |
|  | ***Enabler*** | | | | |
|  |  | Opportunity to form (peer support) groups | *Environmental context and resources: (Resources/material resources)* | Environmental restructuring and Enablement | 1.1 Goal setting (behavior); 1.2 Problem solving; 3.1 Social support (unspecified); 3.2 Social support (practical); 3.3 Social support (emotional); 7.1 Prompts/cues; and 12.1 Restructuring the physical environment |
| ***Social opportunity*** | | | | | |
|  | ***Barrier*** | | | | |
|  |  | Fear of judgment from others | *Social influences: (Social pressure)* | Environmental restructuring and Enablement | 3.1 Social support (unspecified); 3.2 Social support (practical); 3.3 Social support (emotional); 12.1 Restructuring the physical environment; and 12.2 Restructuring the social environment |
|  |  | Fear of disclosure/exposure | *Social influences: (Alienation)* | Environmental restructuring and Enablement | 3.1 Social support (unspecified); 3.2 Social support (practical); 3.3 Social support (emotional); 12.1 Restructuring the physical environment; and 12.2 Restructuring the social environment |
|  |  | Stigma of the condition from others | *Social influences: (Social pressure)* | Environmental restructuring and Enablement | 3.1 Social support (unspecified); 3.2 Social support (practical); 3.3 Social support (emotional); 12.1 Restructuring the physical environment; and 12.2 Restructuring the social environment |
|  |  | HCPs as gatekeepers | *Social/professional role and Identity: (Professional role)* | Environmental restructuring and Enablement | 3.1 Social support (unspecified); 3.2 Social support (practical); 3.3 Social support (emotional); 12.1 Restructuring the physical environment; and 12.2 Restructuring the social environment |
|  | ***Enabler*** | | | | |
|  |  | Social support is desired | *Social influences: (Social support)* | Environmental restructuring and Enablement | 3.1 Social support (unspecified); 3.2 Social support (practical); 3.3 Social support (emotional); 12.1 Restructuring the physical environment; and 12.2 Restructuring the social environment |

^a^COM-B: Capability, Opportunity, Motivation-Behavior.

^b^BCT: behavior change techniques.

^c^SM: self-management.

^d^HCP: health care professional.

*Italics - Theoretical Domains Framework v2 domains
