# Supplementary material for: A Facilitated Web-Based Self-Management Tool for People With Type 1 Diabetes Using an Insulin Pump: Intervention Development Using the Behavior Change Wheel and Theoretical Domains Framework
Source: J Med Internet Res. 2020 May 1;22(5):e13980. doi: 10.2196/13980 (PMC7229530; doi:10.2196/13980)
Supplement: Multimedia Appendix 2 [file jmir_v22i5e13980_app2.docx]

**Multimedia Appendix 2** Matrix of links among the Capability, Opportunity, Motivation-Behavior model, Theoretical Domains Framework domains, intervention functions, and behavior change techniques for health care professionals.

| Behavioral diagnosis using COM-B^a^—barriers and enablers (*stage 1a*) | | | Theoretical Domains Framework v2 domains linking to COM-B components (*stage 1b*) | | Potential intervention functions (*stage 2*) | | BCTs^b^ (using the 93 BCT taxonomy v2; *stage 3*) | |  |
| --- | --- | --- | --- | --- | --- | --- | --- | --- | --- |
| **Psychological capability** | | | | | | | | |  |
|  | **Enabler** | | | | | | | |  |
|  |  | Important to know how and to be confident in using the SM^c^ intervention and how to address psychosocial needs of patients | | *Beliefs about capabilities: (Self-confidence and perceived competence)* | | Education and Training | | 4.1 Instruction on how to perform a behavior; 6.1 Demonstration of the behavior; 8.1 Behavioral practice/rehearsal; and 15.1 Verbal persuasion about Capability | |
| **Physical capability** | | | | | | | | | |
|  | **Enabler** | | | | | | | | |
|  |  | Need training to facilitate the SM tool | | *Physical skills: (Skills development)* | | Training and Enablement | | 1.2 Problem solving; 4.1 Instruction on how to perform a behavior; 6.1 Demonstration of the behavior; and 8.1 Behavioral practice/rehearsal | |
| **Reflective motivation** | | | | | | | | | |
|  | **Barrier** | | | | | | | | |
|  |  | Other/competing priorities in the clinic | | *Goals: (Goal priority)* | | Coercion; Education; Persuasion; and Incentivization | | 1.1 Goal setting (behavior); 1.4 Action planning; 1.5 Review behavior goal(s); 1.8 Behavioral contract; 1.9 Commitment; 4.4 Behavioral experiments; 5.1 Information about health consequences; 5.5 Information about social and environmental consequences; 6.2 Social comparison; 6.3 Information about others’ approval; 7.1 Prompts/cues; 8.1 Behavioral practice/Rehearsal; 9.1 Credible source; 9.3 Comparative imagining of future outcomes; 13.2 Framing/reframing; and 13.3 Incompatible beliefs | |
|  |  | “Is there room for indulgence in the National Health Service?” | | *Optimism: (Pessimism)* | | Coercion; Education; Persuasion; and Incentivization | | 1.1 Goal setting (behavior); 1.4 Action planning; 1.5 Review behavior goal(s); 1.8 Behavioral contract; 1.9 Commitment; 4.4 Behavioral experiments; 5.1 Information about health consequences; 5.5 Information about social and environmental consequences; 6.2 Social comparison; 6.3 Information about others’ approval; 7.1 Prompts/cues; 8.1 Behavioral practice/Rehearsal; 9.1 Credible source; 9.3 Comparative imagining of future outcomes; 13.2 Framing/reframing; and 13.3 Incompatible beliefs | |
|  |  | Potential for peers to “give bad advice,” “What you don't want to be doing is creating problems for problems sake.” | | *Beliefs about consequences: (Outcome expectancies); 3Social/professional role and Identity: (Professional boundaries)* | | Coercion; Education; Persuasion; and Incentivization | | 1.1 Goal setting (behavior); 1.4 Action planning; 1.5 Review behavior goal(s); 1.8 Behavioral contract; 1.9 Commitment; 4.4 Behavioral experiments; 5.1 Information about health consequences; 5.5 Information about social and environmental consequences; 6.2 Social comparison; 6.3 Information about others’ approval; 7.1 Prompts/cues; 8.1 Behavioral practice/Rehearsal; 9.1 Credible source; 9.3 Comparative imagining of future outcomes; 13.2 Framing/reframing; and 13.3 Incompatible beliefs | |
|  |  | Bombarded with *risks and dangers* and professional responsibility | | *Social/professional role and Identity: (Professional role)* | | Coercion; Education; Persuasion; and Incentivization | | 1.1 Goal setting (behavior); 1.4 Action planning; 1.5 Review behavior goal(s); 1.8 Behavioral contract; 1.9 Commitment; 4.4 Behavioral experiments; 5.1 Information about health consequences; 5.5 Information about social and environmental consequences; 6.2 Social comparison; 6.3 Information about others’ approval; 7.1 Prompts/cues; 8.1 Behavioral practice/Rehearsal; 9.1 Credible source; 9.3 Comparative imagining of future outcomes; 13.2 Framing/reframing; and 13.3 Incompatible beliefs | |
|  |  | “Is it (SM) even *our* role?” | | *Environmental context and Resources: (Facilitator)* | | Coercion; Education; Persuasion; and Incentivization | | 1.1 Goal setting (behavior); 1.4 Action planning; 1.5 Review behavior goal(s); 1.8 Behavioral contract; 1.9 Commitment; 4.4 Behavioral experiments; 5.1 Information about health consequences; 5.5 Information about social and environmental consequences; 6.2 Social comparison; 6.3 Information about others’ approval; 7.1 Prompts/cues; 8.1 Behavioral practice/Rehearsal; 9.1 Credible source; 9.3 Comparative imagining of future outcomes; 13.2 Framing/reframing; and 13.3 Incompatible beliefs | |
|  | **Enabler** | | | | | | | | |
|  |  | Social support is relevant for patient group | | *Environmental context and Resources:* (Facilitator) | | Coercion; Education; Persuasion; and Incentivization | | 1.1 Goal setting (behavior); 1.4 Action planning; 1.5 Review behavior goal(s); 1.8 Behavioral contract; 1.9 Commitment; 4.4 Behavioral experiments; 5.1 Information about health consequences; 5.5 Information about social and environmental consequences; 6.2 Social comparison; 6.3 Information about others’ approval; 7.1 Prompts/cues; 8.1 Behavioral practice/Rehearsal; 9.1 Credible source; 9.3 Comparative imagining of future outcomes; 13.2 Framing/reframing; and 13.3 Incompatible beliefs | |
|  |  | (GENIE) Fits in with clinic | | *Environmental context and Resources: (Organizational culture/climate)* | | Coercion; Education; Persuasion; and Incentivization | | 1.1 Goal setting (behavior); 1.4 Action planning; 1.5 Review behavior goal(s); 1.8 Behavioral contract; 1.9 Commitment; 4.4 Behavioral experiments; 5.1 Information about health consequences; 5.5 Information about social and environmental consequences; 6.2 Social comparison; 6.3 Information about others’ approval; 7.1 Prompts/cues; 8.1 Behavioral practice/Rehearsal; 9.1 Credible source; 9.3 Comparative imagining of future outcomes; 13.2 Framing/reframing; and 13.3 Incompatible beliefs | |
| **Automatic motivation** | | | | | | | | | |
|  | **Barrier** | | | | | | | | |
|  |  | Fear backlash/responsibility | | *Emotion: (Negative affect)* | | Enablement; Environmental restructuring; and Modeling (for other clinics) | | 12.1 Restructuring the physical environment; 12.2 Restructuring the social environment; 13.3 Incompatible beliefs | |
|  | **Enabler** | | | | | | | | |
|  |  | Like GENIE^d^ and have seen how much patients benefit from peer and other holistic support | | *Emotion: (Positive affect)* | | Enablement; Environmental restructuring; and Modeling (for other clinics) | | 12.1 Restructuring the physical environment; 12.2 Restructuring the social environment; and 13.3 Incompatible beliefs | |
| **Physical opportunity** | | | | | | | | | |
|  | **Barrier** | | | | | | | | |
|  |  | Lack of time to undertake/facilitate further SM support in clinic | | *Environmental context and Resources: (Barrier)* | | Enablement and Environmental restructuring | | 1.2 Problem solving; 3.1 Social support (unspecified); 12.1 Restructuring the physical environment; and 12.2 Restructuring the social environment | |
|  |  | Lack of capacity in the clinic | | *Environmental context and Resources: (Resources/material resources)* | | Enablement and Environmental restructuring | | 1.2 Problem solving; 3.1 Social support (unspecified); 12.1 Restructuring the physical environment; and 12.2 Restructuring the social environment | |
|  |  | Lack of a holistic support provision in the organizational culture | | *Environmental context and Resources: (Organizational culture/climate)* | | Enablement and Environmental restructuring | | 1.2 Problem solving; 3.1 Social support (unspecified); 12.1 Restructuring the physical environment; and 12.2 Restructuring the social environment | |
|  |  | “The lost tribe” (patients clinics cannot reach) | | *Environmental context and Resources: (Barriers)* | | Enablement and Environmental restructuring | | 1.2 Problem solving; 3.1 Social support (unspecified); 12.1 Restructuring the physical environment; and 12.2 Restructuring the social environment | |
|  | **Enabler** | | | | | | | | |
|  |  | Patient time restraints for SM/to engage with clinic | | *Environmental context and Resources: (Person × environment interaction)* | | Enablement and Environmental restructuring | | 1.2 Problem solving; 3.1 Social support (unspecified); 12.1 Restructuring the physical environment; and 12.2 Restructuring the social environment | |
|  |  | Using leaflets to advertise SM intervention | | *Environmental context and Resources: (Facilitators)* | | Enablement and Environmental restructuring | | 1.2 Problem solving; 3.1 Social support (unspecified); 12.1 Restructuring the physical environment; and 12.2 Restructuring the social environment | |
|  |  | Clinics desire/want to be flexible and want to offer more direct access for SM/to peer support | | *Environmental context and Resources: (Facilitator)* | | Enablement and Environmental restructuring | | 1.2 Problem solving; 3.1 Social support (unspecified); 12.1 Restructuring the physical environment; and 12.2 Restructuring the social environment | |
| **Social opportunity** | | | | | | | | | |
|  | **Enabler** | | | | | | | | |
|  |  | Clinicians stress that their patients often want to speak to other patients | | *Social influences: (Social support)* | | Enablement and Environmental restructuring | | 12.1 Restructuring the physical environment and 12.2 Restructuring the social environment | |

^a^COM-B: Capability, Opportunity, Motivation-Behavior.

^b^BCT: behavior change techniques.

^c^SM: self-management.

^d^GENIE: Generating Engagement in Networks InvolvEment.

*Italics - Theoretical Domains Framework v2 domains
